# Comprehensive Transcriptomic Analysis Identifies Novel Antiviral Factors Against Influenza A Virus Infection

**DOI:** 10.3389/fimmu.2021.632798

**Published:** 2021-07-21

**Authors:** Ao Zhou, Xia Dong, Mengyun Liu, Bin Tang

**Affiliations:** ^1^ College of Animal Science and Nutritional Engineering, Wuhan Polytechnic University, Wuhan, China; ^2^ Basic Medical College, Southwest Medical University, Luzhou, China; ^3^ Key Lab of Process Analysis and Control of Sichuan Universities, Yibin University, Yibin, China

**Keywords:** gene express profile, Influenza A virus, immune response, BATF2, HERC5

## Abstract

Influenza A virus (IAV) has a higher genetic variation, leading to the poor efficiency of traditional vaccine and antiviral strategies targeting viral proteins. Therefore, developing broad-spectrum antiviral treatments is particularly important. Host responses to IAV infection provide a promising approach to identify antiviral factors involved in virus infection as potential molecular drug targets. In this study, in order to better illustrate the molecular mechanism of host responses to IAV and develop broad-spectrum antiviral drugs, we systematically analyzed mRNA expression profiles of host genes in a variety of human cells, including transformed and primary epithelial cells infected with different subtypes of IAV by mining 35 microarray datasets from the GEO database. The transcriptomic results showed that IAV infection resulted in the difference in expression of amounts of host genes in all cell types, especially those genes participating in immune defense and antiviral response. In addition, following the criteria of *P*<0.05 and |logFC|≥1.5, we found that some difference expression genes were overlapped in different cell types under IAV infection *via* integrative gene network analysis. IFI6, IFIT2, ISG15, HERC5, RSAD2, GBP1, IFIT3, IFITM1, LAMP3, USP18, and CXCL10 might act as key antiviral factors in alveolar basal epithelial cells against IAV infection, while BATF2, CXCL10, IFI44L, IL6, and OAS2 played important roles in airway epithelial cells in response to different subtypes of IAV infection. Additionally, we also revealed that some overlaps (BATF2, IFI44L, IFI44, HERC5, CXCL10, OAS2, IFIT3, USP18, OAS1, IFIT2) were commonly upregulated in human primary epithelial cells infected with high or low pathogenicity IAV. Moreover, there were similar defense responses activated by IAV infection, including the interferon-regulated signaling pathway in different phagocyte types, although the differentially expressed genes in different phagocyte types showed a great difference. Taken together, our findings will help better understand the fundamental patterns of molecular responses induced by highly or lowly pathogenic IAV, and the overlapped genes upregulated by IAV in different cell types may act as early detection markers or broad-spectrum antiviral targets.

## Introduction

Influenza A virus (IAV) infection causes severe respiratory symptoms and persistent morbidity as well as mortality during annual seasonal or pandemic outbreaks, resulting in a severe threat to public health and safety, and even huge economic burden (1). Over the past decade, influenza outbreaks and pandemics have been caused by different subtypes of IAV, including H1N1, H3N2, swine-origin H1N1, and highly pathogenic avian influenza viruses (2–4), suggesting that the deeper biologic and epidemiologic mechanisms should be revealed to confidently and accurately predict the next influenza outbreak. Accumulative evidence has shown that IAV is capable of eliciting cellular immune response thought changing the expression of multiple genes in diverse types of cells, which in turn inhibit IAV infection. Airway epithelial cells are the preferred location for IAV replication and dissemination, and IAV infection induced toll-like receptors (TLRs)-related genes expression in responses to the pathogen (5). Moreover, other cell types, including endothelial cells, macrophages, monocytes, dendritic cells, and neutrophils, play important roles in response to IAV infection (6–10). During IAV infection, interferon, interferon-stimulated genes, and cytokines were secreted and activated in epithelial cells and immune cell types such as macrophages, monocytes, and neutrophils to facilitate antiviral responses. However, IAV could also elicit inflammation and cause various disorders of the respiratory system. Therefore, the systematic comparison of host responses of various types of cells to a range of strains of IAV still need to be further investigated.

Microarray technology with maturity is a powerful tool for the global view of gene expression levels, and enormous amounts of genome-wide gene expression microarray studies were distributed and archived in the gene expression omnibus (GEO) repository at the National Centre for Biotechnology Information (NCBI) in the last few decades, providing the chance for investigators revisiting these data to solve scientific questions. In this current study, we collected various transcriptomic datasets that were involved in diverse types of cells infected with subtypes of IAV, in order to examine common aspects of host cell responses to various subtypes of IAV infection. By integrating the global gene expression data, our results suggested that although the differentially expressed genes involved in host responses might not conform, the similar immune responses of diverse cell types were triggered by the infection of different subtypes of IAV.

## Materials and Methods

### Data Preparation

The public gene expression resource of human cells infected with different IAV subtypes are mainly collected from the GEO database (https://www.ncbi.nlm.nih.gov/geo/). Totally, after searching for keywords related to IAV, we selected 35 data series about IAV for this research (**Tables 1**–**3**, and **5**). The normalization of data of target profiles from the GEO database was performed using the limma package (30) to detect differentially expressed genes (DEGs). Then, significant DEGs were obtained using this set of parameters: P value < 0.05 and abs [log fold change (logFC)] > 1.5.

**Table 1:** The details of gene expression profiles on A549 cells from the GEO database.

GEO_no.	Platforms	Cells	Strains	Hours post-infection (Hpi)
GSE31470 (11, 12)	GPL570	A549	A/WSN/33 (H1N1)	10 hpi
GSE31471 (11, 12)	GPL570	A549	A/Duck/Malaysia/01 (H9N2)	10 hpi
GSE31472 (11, 12)	GPL570	A549	A/Duck/Malaysia/F118/08/2004 (H5N2)	10 hpi
GSE31473 (11, 12)	GPL570	A549	A/Duck/Malaysia/F59/04/1998 (H5N2)	10 hpi
GSE31474 (11, 12)	GPL570	A549	A/Duck/Malaysia/F189/07/2004 (H5N2)	10 hpi
GSE31475 (11, 12)	GPL570	A549	A/Duck/Malaysia/F119/3/1997 (H5N3)	10 hpi
GSE32878 (13)	GPL14715	A549	A/WSN/33 (H1N1)	10 hpi
GSE31518 (11, 12)	GPL570	A549	A/Singapore/478/2009 (H1N1)	10 hpi
GSE58741 (14)	GPL17077	A549	A/WSN/33 (H1N1)	12 hpi
GSE106279 (15)	GPL17586	A549	A/Puerto Rico/8/1934 (H1N1)	24 hpi

**Table 2:** The details of gene expression profiles on Calu-3 cells from the GEO database.

GEO_no.	Platforms	Cells	Strains	Hours post-infection (Hpi)
GSE19580 (16)	GPL8432	Calu-3	A/Sharp-Tailed Sandpiper/Australia/6/2004 (H11N9)	6 and 24 h
GSE28166 (17)	GPL6480	Calu-3	A/VN/1203/04 (H5N1)	7, 12, and 24 h
GSE33142 (18)	GPL6480	Calu-3	A/VN/1203/04 (H5N1)	7, 12, and 24 h
GSE37571 (19)	GPL6480	Calu-3	A/CA/04/2009 (H1N1)	7, 12, and 24 h
GSE40844 (17, 19)	GPL6480	Calu-3	A/Netherlands/602/2009 (H1N1); A/California/04/2009 (H1N1)	7, 12, and 24 h 7, 12, and 24 h
GSE49840 (20)	GPL17077	Calu-3	A/Anhui/01/2013 (H7N9); A/Netherland/219/2003 (H7N7); A/Vietnam/1203/2004 (H5N1); A/Panama/2007/1999 (H3N2)	7, 12, and 24 h
GSE80697 (21)	GPL13497	Calu-3	A/California/04/2009 (H1N1)	7, 12, and 24 h

**Table 3:** The details of gene expression profiles on epithelial and endothelial cells from the GEO database.

GEO_no.	Platforms	Cells	Strains	Hours post-infection (Hpi)
GSE19392 (22)	GPL3921	Human bronchial epithelial cells (HBECs)	A/PR/8/34 (H1N1)	18 h
GSE24533 (23)	GPL6244	Human type I-like alveolar epithelial cells	A/HK/415742/2009 (H1N1); A/HK/54/1998 (H1N1)	8 h
GSE30723 (24)	GPL570	Human type II-like alveolar epithelial cells	A/PR/8/34 (H1N1)	24 h
GSE41475 (25)	GPL16163	Primary human airway epithelial cells	CA09 (H1N1); TN09 (H1N1); BSB07 (H1N1); IT95 (H1N1)	24 h
GSE48466 (26)	GPL570	Well-differentiated primary human bronchial epithelial cells (wd-NHBE)	A/BN/59/07 (H1N1); A/KY/180/10 (H1N1); A/KY/136/10 (H1N1)	36 h
GSE65699 (27)	GPL10558	Human retinal pigment epithelium cell line (RPE)	A/WSN/33 (H1N1)	10 h
GSE13637 (28)	GPL570	Human umbilical vein endothelial cells (HUVEC)	PR8 (H1N1); FPV (H7N7); H5N1	5 h
GSE59226 (29)	GPL570	Human umbilical vein endothelial cells (HUVEC)	H9N2	24 h

### Overlap Genes and Functional Enrichment Analysis

DEGs with abs (logFC) > 1.5 and P value < 0.05 from each data series were obtained to analyze the overlap genes. Then, Gene Ontology (GO) and Kyoto Encyclopedia of Genes and Genomes (KEGG) biological pathway analyses were performed to predict the functionalities of differentially expressed overlap genes using the R package clusterProfiler (31). In this study, a Venn diagram, heatmap, and volcano plot were constructed using R language and R packages, including VennDiagram (32), ggplot2 (33), and pheatmap (34).

## Results

### Host Transcriptional Response to Influenza Virus Infection on Human Lung Epithelial Cells (A549)

**Figure 1:**
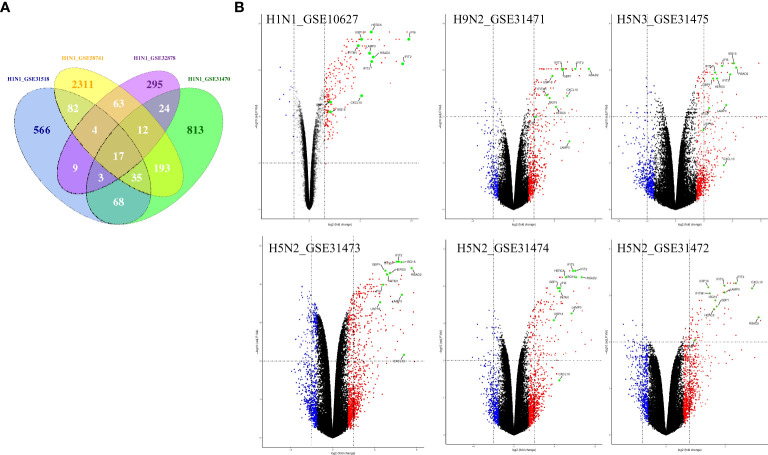
The common gene expression in A549 cells infected with IAV. **(A)** The Venn diagram shows the overlapping genes in 4 H1N1-infected datasets, 17 genes were common to the 4 H1N1-infected groups; **(B)** Volcano plots showing differentially expressed genes for H1N1, H9N2, H5N3, and H5N2-infected A549 cells. The vertical dashed lines correspond to 1.5-fold up and down expression, and the horizontal dashed line represents a *p-*value of 0.01. Blue and red dots indicate significantly downregulated and upregulated genes, respectively. Genes that were not classified as differentially expressed are plotted in black. The 11 overlapped genes identified in different IAV subtype infection are exhibited in the Volcano plots.

To illustrate host cell response to influenza virus infection, the global gene expression profiles from four cell-based time-series gene expression datasets in A549, a lung epithelial cell known to be highly susceptible to IAV, were analyzed (**Table 1**) (11–15). Matched with the criteria of *P*<0.05 and |logFC|≥1.5, the Venn diagram showed that 17 genes (IFI6, IFIT2, HERC5, ISG15, RSAD2, GBP1, IFIT3, IFNB1, IFITM1, LAMP3, USP18, CXCL10, IER5L, HAMP, MX2, FERMT3, and ZC3HAV1) were differentially expressed genes (DEGs) and overlapped in these different databases (**Figure 1A**). Then, to further identify whether the mRNA expression of these genes had the same expression pattern in different subtypes of influenza virus infection, the databases with the other subtypes of influenza virus were analyzed and the result showed that the mRNA expression of 11 overlapped genes (IFI6, IFIT2, ISG15, HERC5, RSAD2, GBP1, IFIT3, IFITM1, LAMP3, USP18, and CXCL10) were also remarkably upregulated in A549 cells infected with A/Puerto Rico/8/1934 (H1N1), H5N2, H5N3, and H9N2 (**Figure 1B**) compared with mock, respectively. Overall, these results indicated that IFI6, IFIT2, ISG15, HERC5, RSAD2, GBP1, IFIT3, IFITM1, LAMP3, USP18, and CXCL10 might play key roles in IAV infection.

### Host Transcriptional Response to Influenza Virus Infection on Human Airway Epithelial Cells (Calu-3)

**Figure 2:**
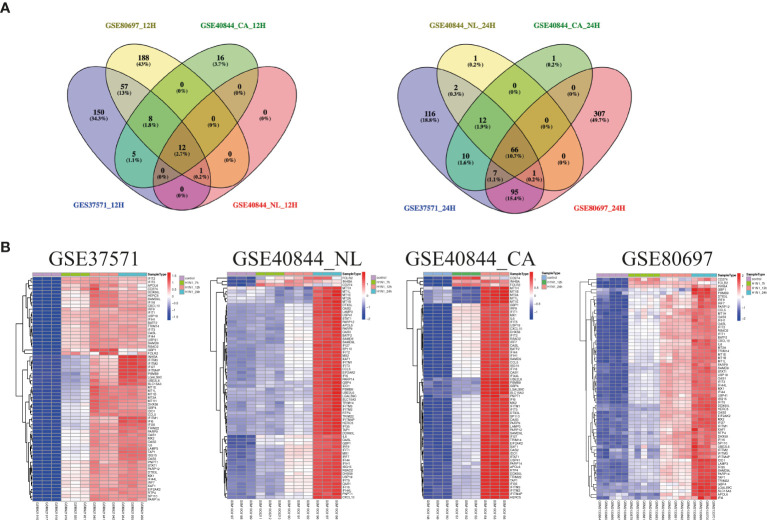
The expression profiling of common genes from four different data series infected with H1N1 in Calu-3 cells. **(A)** Venn diagram of the overlap genes between different data with 12 hpi (left) or 24 hpi (right). Twelve genes were common to the four H1N1-infected Calu-3 cells at 12 hpi, while 66 overlapped genes were found at 24 hpi. **(B)** Heatmap of differentially expressed patterns of the overlap genes in IAV-infected Calu-3 cells. Row represents the overlap genes and each column corresponds to a sample. NL: A/Netherland/219/2003, CA: A/California/04/2009.

**Figure 3:**
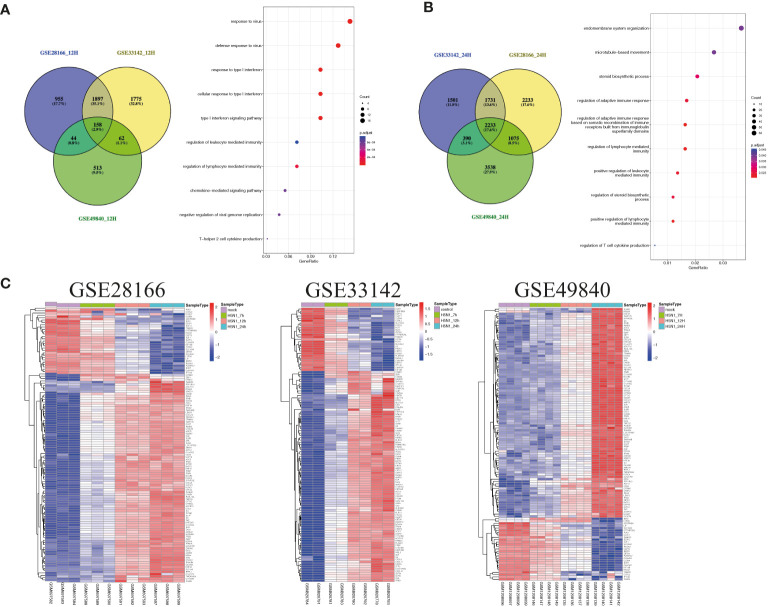
The common gene expression from three different data series in Calu-3 cells infected with H5N1. **(A)** A Venn diagram of the overlap genes between different data with 12 hpi. A total of 158 genes were common to the four H5N1-infected Calu-3 cells at 12 hpi; KEGG pathways of differentially expressed overlap genes. The vertical axis shows the number of genes, and the horizontal axis shows the GeneRatio. **(B)** A Venn diagram of the overlap genes between different data with 24 hpi. In total, 2233 overlapped genes were common to the four H5N1-infected Calu-3 cells at 24 hpi. KEGG pathways of differentially expressed overlap genes. The vertical axis shows the number of genes, and the horizontal axis shows the GeneRatio. **(C)** Heatmap of differentially expressed patterns of the overlap genes in IAV-infected Calu-3 cells. Row represents the overlap genes and each column corresponds to a sample.

**Figure 4:**
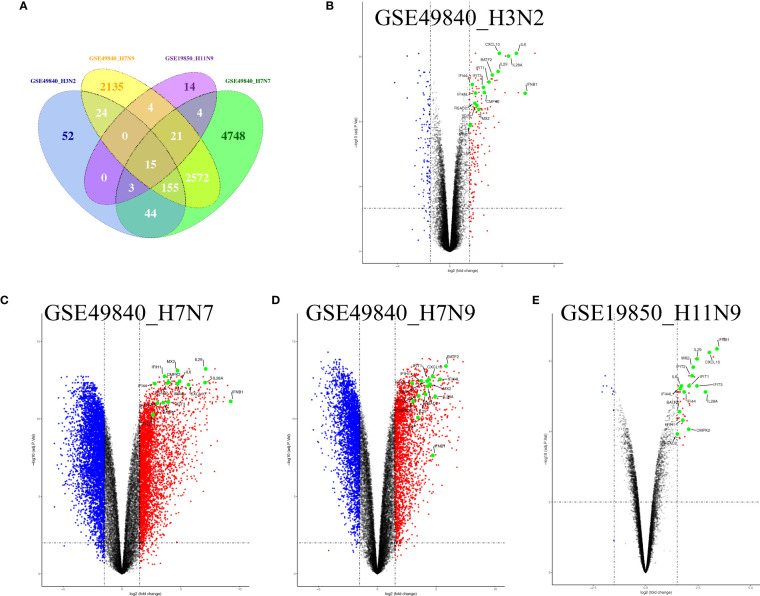
Fifteen overlap genes were identified to significantly upregulate in H3N2, H7N7, H7N9, and H11N9-infected Calu-3 cells. **(A)** Venn diagram of the overlap genes; **(B)** Volcano plots of the overlaps between mock and H3N2-infected cells; blue and red dots indicate significantly downregulated and upregulated genes, respectively. **(C)** Volcano plots of the overlaps between mock and H7N7-infected cells; blue and red dots indicate significantly downregulated and upregulated genes, respectively. **(D)** Volcano plots of the overlaps between mock and H7N9-infected cells; blue and red dots indicate significantly downregulated and upregulated genes, respectively. **(E)** Volcano plots of the overlaps between mock and H11N9-infected cells. The vertical dashed lines correspond to 1.5-fold up and down expression and the horizontal dashed line represents a *p*-value of 0.01. Blue and red dots indicate significantly downregulated and upregulated genes, respectively. The 15 overlapped genes identified in different IAV subtype infection are exhibited in the Volcano plots.

Airway epithelial cells are key to regulate pulmonary inflammatory and immune responses against influenza virus challenges. To acquire the knowledge on how airway epithelial cells contribute to the defense against IAV infection, the data from Calu-3 cells infected with influenza virus strains (H1N1, H3N2, H5N1, H7N7, H7N9, and H11N9) were collected and analyzed (**Table 2**) (16–21). In the Calu-3 cells infected with H1N1, the results from four transcriptomic datasets (GSE80697, GSE40844_CA, GSE40844_NL, and GSE37571) showed that twelve overlapping DEGs containing USP18, RSAD2, IFIT2, OAS1, MX1, IFI44, MX2, IFIT1, DHX58, IFITM1, IFI44L, and FOLR2 were identified to have a higher expression compared with the uninfected cells at 12 h post-infection (hpi). In addition, at 24 hpi, there were 66 overlapping DEGs (**Supplementary File 1**) significantly regulated by IAV (**Figure 2A**), according to the criteria of *P*<0.05 and |logFC|≥1.5. Interestingly, all of those 12 common DEGs at 12 hpi were also significantly upregulated by IAV infection at 24 hpi. In addition, hierarchical clustering analysis was used to assess the expression profiling of the common DEGs in different samples with different infection time-points in four H1N1 infection data series, and heatmap diagrams revealed that a large proportion of the overlapping DEGs mRNA expression exhibited an infection time-dependent upregulation (**Figure 2B**). Subsequently, host transcriptome profiles in Calu-3 cells infected with H5N1 from GSE28166, GSE33142, and GSE49840 were analyzed. The results showed that a total of 158 overlapping DEGs were identified in Calu-3 cells at 12 hpi, while there were 2233 overlapping DEGs at 24 hpi upon H5N1 strains infection following the criteria of *P*<0.05 and |logFC|≥1.5 (**Figures 3A, B**). In addition, KEGG pathway analysis showed that the overlapping genes were mainly concentrated on type I interferon-mediated signaling and host response to the virus at 12 hpi, while the genes related with the steroid biosynthetic process and adaptive immune response were enriched in H5N1-infected Calu-3 cells at 24 hpi (**Figures 3A, B**). Moreover, hierarchical clustering analysis showed that a large proportion of the overlapping DEGs mRNA expression exhibited an infection time-dependent upregulation in three H5N1 infection data series (**Figure 3C**).Additionally, H3N2, H7N7, H7N9, and H11N9-induced gene expression profiles at 24 hpi were overlapped to identify 15 upregulated genes (IFNB1, IL6, CMPK2, IL28A, IFIT3, IL29, RSAD2, MX2, IFI44, IFIT2, IFIH1, IFIT1, IFI44L, CXCL10, BATF2) following the criteria of *P*<0.05 and |logFC|≥1.5 (**Figures 4A–E**). Furthermore, integration of the overlapping DEGs regulated with the six subtypes of influenza virus at 24 hpi identified eight overlapped genes (BATF2, IFNB1, IL28A, IL29, IFIT2, CXCL10, IFI44L, and IL6) that were significantly overexpressed in IAV-infected Calu-3 cells.

### Host Transcriptional Response to Influenza Virus Infection on Human Primary Epithelial Cells and Endothelial Cells

**Figure 5:**
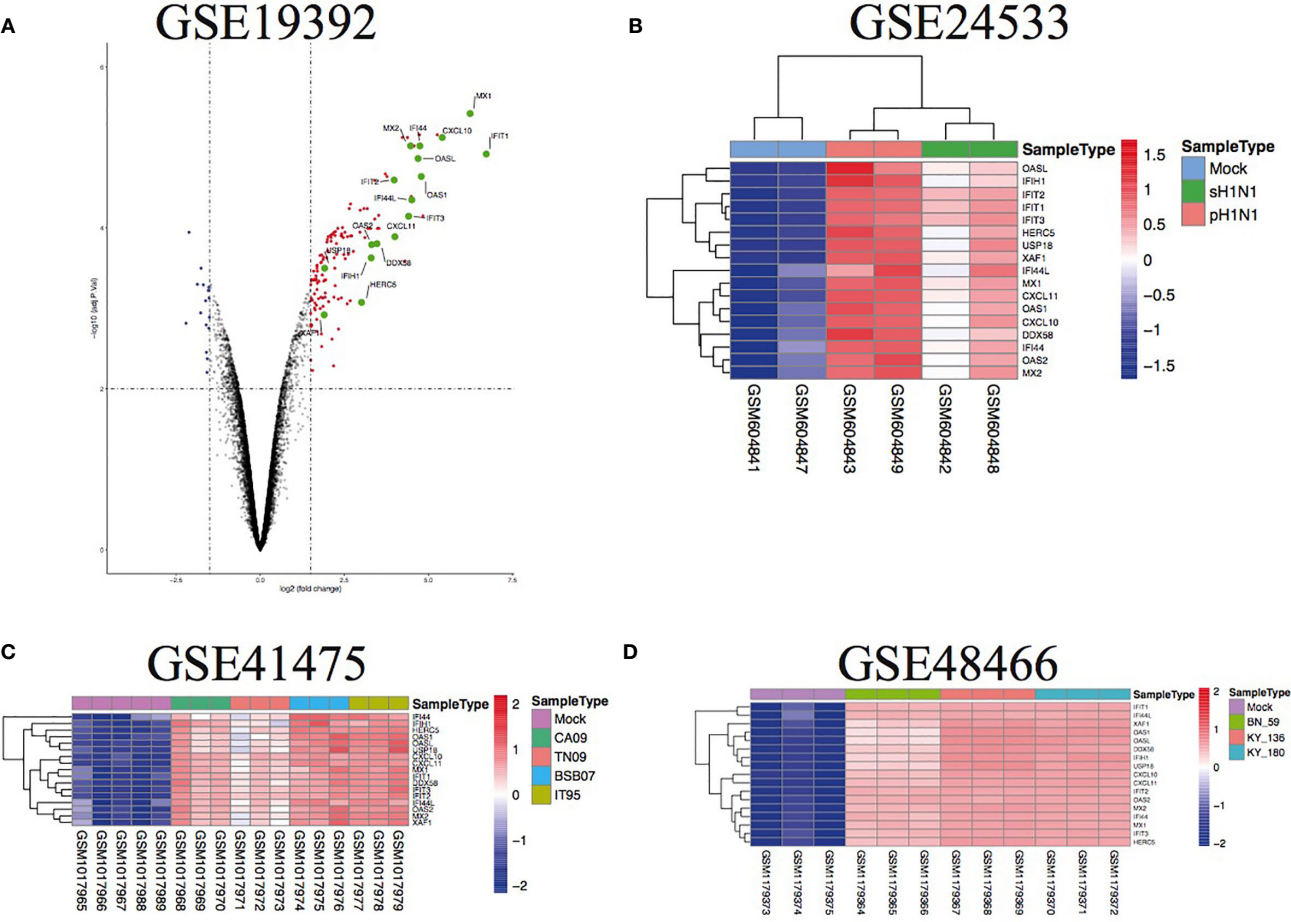
The overlap regulation gene expression of diverse primary epithelial cells in response to H1N1 infection. **(A)** Volcano plots of the overlaps between mock and H1N1-infected human bronchial epithelial cells. The vertical dashed lines correspond to 1.5-fold up and down expression and the horizontal dashed line represents a *p*-value of 0.01. Blue and red dots indicate significantly downregulated and upregulated genes, respectively. The 17 overlapped genes identified in different IAV subtype infections are exhibited in the Volcano plots. **(B)** Heatmap of the overlaps in mock or H1N1-infected human type I-like alveolar epithelial cells. Row represents the overlap genes and each column corresponds to a sample; **(C)** heatmap of the overlaps in mock or H1N1-infected primary human airway epithelial cells. Row represents the overlap genes and each column corresponds to a sample; **(D)** heatmap of the overlaps in mock or H1N1-infected well-differentiated primary human bronchial epithelial cells. Row represents the overlap genes and each column corresponds to a sample.

**Figure 6:**
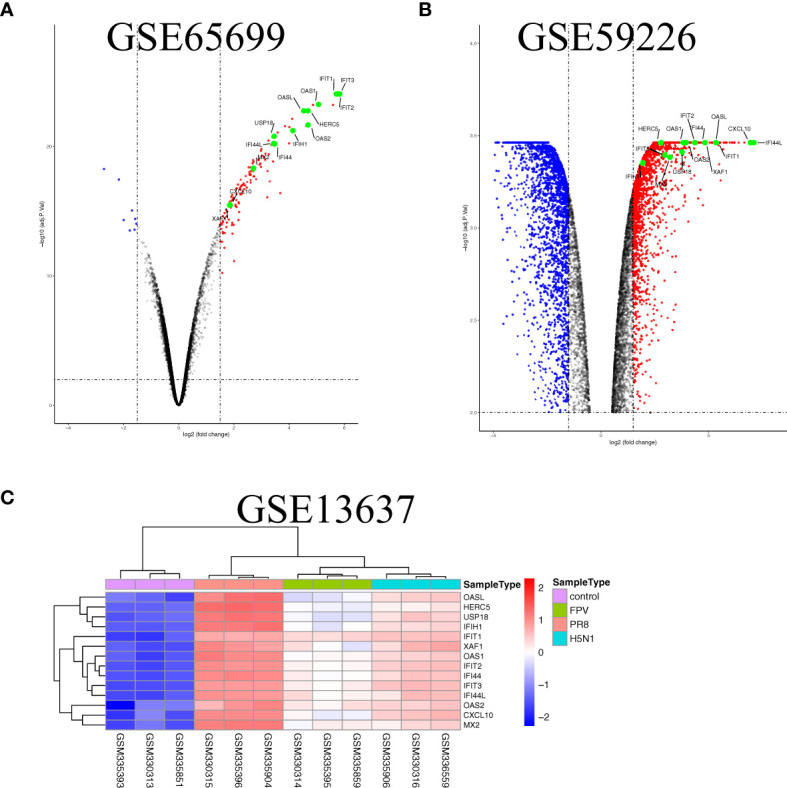
The overlap regulation gene expression of epithelium and endothelial cells lines in response to IAV infection. **(A)** Volcano plots of the overlaps between mock and the H1N1-infected human retinal pigment epithelium cell line. The vertical dashed lines correspond to 1.5-fold up and down expression and the horizontal dashed line represents a *p*-value of 0.01. Blue and red dots indicate significantly downregulated and upregulated genes, respectively. The 13 overlapped genes identified in different IAV subtype infections are exhibited in the Volcano plots. **(B)** Volcano plots of the overlaps between mock and H9N2-infected human umbilical vein endothelial cells. The vertical dashed lines correspond to 1.5-fold up and down expression and the horizontal dashed line represents a *p*-value of 0.01. Blue and red dots indicate significantly downregulated and upregulated genes, respectively. The 13 overlapped genes identified in different IAV subtype infections are exhibited in the Volcano plots. **(C)** Heatmap of the overlaps in mock or H1N1, H5N1, and H7N7-infected human umbilical vein endothelial cells. Row represents the overlap genes and each column corresponds to a sample.

To further illustrate the effect of transcriptome alteration in influenza virus infection on human respiratory tract epithelial cells, we analyzed gene expression profiles of influenza virus-infected primary epithelial cells (**Table 3**) (22–29), including primary human bronchial epithelial cells (HBEC), well-differentiated human bronchial epithelial cells (wd-NHBE), human primary airway epithelial cell, human type I-like alveolar epithelial cells, and human type II-like alveolar epithelial cells, all of which were infected by H1N1 influenza. As above, matching the criteria of *P*<0.05 and |logFC|≥1.5, in total 17 overlapping genes (IFI44L, IFI44, HERC5, OASL, CXCL10, MX2, CXCL11, OAS2, XAF1, IFIT3, USP18, IFIH1, OAS1, DDX58, MX1, IFIT2, and IFIT1) were identified to be significantly increased with influenza virus infection in all human primary epithelial cells (**Figures 5A–D**) and these commonly responsive genes were largely related with interferon-stimulated response. To identify whether other strains of influenza virus also induced the same response in other epithelium and endothelial cells lines, human retinal pigment epithelium cell line (RPE) and human umbilical vein endothelial cells (HUVEC) were infected with other subtypes of influenza virus (H7N7, H5N1, and H9N2) and the genome-wide gene expression pattern of infected cells was analyzed. The results showed that 14 of 17 overlapping genes (IFI44L, IFI44, HERC5, OASL, CXCL10, MX2, OAS2, XAF1, IFIT3, USP18, IFIH1, OAS1, IFIT2, and IFIT1) in primary epithelial cells were remarkably upregulated in H1N1, H7N7, H5N1, and H9N2-infected RPE or HUVEC (**Figures 6A–C**), indicating that these overlapped DEGs may play key roles in regulating epithelium cells response against IAV infection. Moreover, we found that BATF2 could be significantly upregulated with H1N1 and other strain infections in all collected datasets of human primary epithelial cells and endothelial cells, expect one dataset (GSE19392) where the BATF2 gene was not included in the microarray platform (**Table 4**). Overall, our results suggested that a similar host response was induced by different influenza strains in human epithelial and endothelial cells, regardless of high or low pathogenicity.

**Table 4:** The mRNA expression of the BATF2 gene infected with influenza virus.

GEO_no.	Cells	Strains	BATF2 mRNA expression (logFC)/P-value
GSE19392	Human bronchial epithelial cells (HBECs)	A/PR/8/34 (H1N1)	No value*
GSE24533	Human type I-like alveolar epithelial cells	A/HK/415742/2009 (H1N1); A/HK/54/1998 (H1N1)	0.82/0.029 1.21/0.004
GSE30723	Human type II-like alveolar epithelial cells	A/PR/8/34 (H1N1)	3.83/3.63E-07
GSE41475	Primary human airway epithelial cells	CA09 (H1N1); TN09 (H1N1); BSB07 (H1N1); IT95 (H1N1)	2.4/1.73E-08 1.75/2.4E-07 2.9/1.67E-10 3.09/9.62E-09
GSE48466	Well-differentiated primary human bronchial epithelial cells (wd-NHBE)	A/BN/59/07 (H1N1); A/KY/180/10 (H1N1); A/KY/136/10 (H1N1)	4/7.94E-13 5.69/3.01E-14 5.38/5.05E-14
GSE65699	Human retinal pigment epithelium cell line (RPE)	A/WSN/33 (H1N1)	2.42/5.06E-21
GSE13637	Human umbilical vein endothelial cells (HUVEC)	A/PR/8/34 (H1N1); A/FPV/Weybridge (H7N7); A/Thailand/1(KAN-1)/2004 (H5N1)	5.22/3.39E-09 3.76/6.83E-08 3.96/4.3E-08
GSE59226	Human umbilical vein endothelial cells (HUVEC)	A/Chicken/Hebei/4/2008 (H9N2)	3.96/1.33E-05

*the BATF2 gene was not present in the microarray platform.

### Host Transcriptional Response to Influenza Virus Infection on Phagocytes

**Figure 7:**
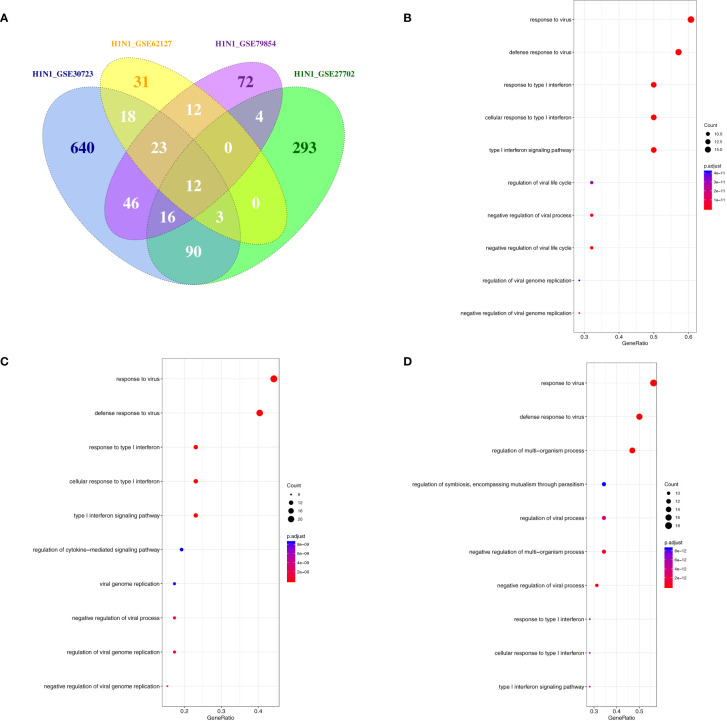
The overlap regulation gene expression of macrophages in response to H1N1 infection. **(A)** A Venn diagram of the overlap genes in H1N1-infected macrophages, 12 genes were common to the two infected groups. **(B)** GO biological processes of differentially expressed overlap in H1N1-infected monocytes. The vertical axis shows the functional classification, and the horizontal axis shows the GeneRatio; **(C)** GO biological processes of differentially expressed overlap in H1N1-infected monocyte-derived DCs. The vertical axis shows the functional classification, and the horizontal axis shows the GeneRatio; **(D)** GO biological processes of differentially expressed overlap in H1N1-infected neutrophils. The vertical axis shows the functional classification, and the horizontal axis shows the GeneRatio.

Phagocytes containing monocytes, macrophages, dendritic cells, and neutrophils are critical in the recognition, engulfment, and destruction of invading pathogens. To uncover the antiviral activities of phagocytes against different subtypes of influenza viruses, the transcriptome analysis of gene expression profiles was performed in human phagocytic cells (PBMC, monocytes, macrophages, pDC, and neutrophils) that were infected with different subtypes of influenza viruses, including H1N1, H5N1, and H7N7 (**Table 5**) (24, 27, 35–41). Following the criteria of *P*<0.05 and |logFC|≥1.5, 12 genes (C19orf66, IL6, HESX1, IFNB1, IFITM3, ISG20, ISG15, HERC5, IFIT1, CCL8, IFIT2, and CXCL10) were overlapped and upregulated in H1N1-infected monocyte-derived macrophages and primary alveolar macrophages (**Figure 7A**). Moreover, BAFT2 was also significantly increased in multiple data series of macrophages, except GSE27702 where the BATF2 gene was not included in the microarray platform. On the side, 29, 54, and 35 overlapped DEGs were significantly regulated in H1N1-infected monocytes, monocyte-derived DCs, and neutrophils, respectively (**Supplementary File 2**), and further Gene Ontology (GO) and Venn diagram analysis showed that H1N1 infection triggered a stronger host response with an elevated expression of cytokines and interferons signaling molecules in phagocytic cells, which in turn blocked IAV infection (**Figures 7B–D**). Five common genes (OASL, IFIT3, RSAD2, HERC5, and IFIT1) identified in macrophages were also found to be upregulated in monocytes, monocyte-derived DCs, and neutrophils infected with H1N1.

**Table 5:** The details of gene expression profiles on phagocytes from the GEO database.

GEO_no.	Platforms	Cells	Strains	Hours post-infection (Hpi)
GSE27702 (35)	GPL571	Monocyte-derived macrophages	A/PR/8/34 (H1N1); A/FPV/Weybridge (H7N7); A/Thailand/1(KAN-1)/2004 (H5N1)	5 h
GSE30723 (24)	GPL570	Primary alveolar macrophages	A/PR/8/34 (H1N1)	4 h
GSE62127 (27)	GPL10558	Monocyte-derived macrophages	A/WSN/33 (H1N1)	8 h
GSE79854 (36)	GPL10558	Monocyte-derived macrophages	A/WSN/33 (H1N1)	6 h
GSE35283 (35)	GPL570	Monocytes	A/PR/8/34 (H1N1); A/FPV/Weybridge (H7N7); A/Thailand/1(KAN-1)/2004 (H5N1)	5 h
GSE35473 (37)	GPL6884	Monocytes	A/PR/8/34 (H1N1)	6 h
GSE41067 (38)	GPL10558	Monocyte-derived DCs	A/New Caledonia/20/1999 (H1N1)	8 h
GSE66486 (39)	GPL10558	PBMC	A/CA/4/2009 (H1N1)	8 h
GSE68849 (40)	GPL10558	Monocyte-derived DCs	A/PR/8/34 (H1N1)	8 h
GSE100865 (41)	GPL16686	Neutrophils	A/Kawasaki/UTK-4/2009 (H1N1); A/Mexico/4108/2009 (H1N1)	6 h

**Figure 8:**
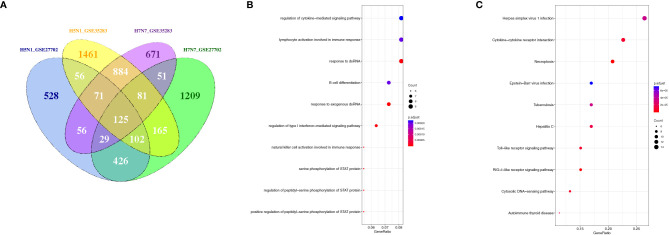
The overlapped regulation gene expression of monocytes and macrophages in response to avian influenza virus infection. **(A)** A Venn diagram of the overlap genes between H5N1 and H7N7-infected monocytes and monocyte-derived macrophages; **(B)** GO biological processes of differentially expressed overlap. The vertical axis shows the functional classification, and the horizontal axis shows the GeneRatio; **(C)** KEGG pathways of differentially expressed overlap genes. The vertical axis shows the number of genes, and the horizontal axis shows the GeneRatio.

In addition, to illustrate whether there was a similar response of phagocytes to other subtypes of influenza A virus infection, the globe gene expression profiles of avian H5N1 and H7N7-infceted monocytes and monocyte-derived macrophages were analyzed. The Venn diagram revealed that a total of 125 different expression genes were found to overlap in the avian flu-infected cells (**Figure 8A**). Moreover, the results of GO and KEGG pathway analysis showed that the overlap genes were mainly concentrated on cytokine-mediated signaling, type I interferon-mediated signaling, defense response to virus, and necroptosis (**Figures 8B, C**). A similar antiviral response, including the type I interferon-mediated signaling pathway, cytokine signaling pathway, and antiviral defense in H1N1-infceted phagocytes was also induced in human phagocytes during highly or lowly pathogenic influenza virus infection, although an overlapped gene was not found in H1N1, H5N1, and H7N7-infceted phagocytes.

## Discussion

Host defense responses elicited by IAV are critical to protect the host against IAV. However, the mechanisms underlying how the host response is activated among IAVs was not fully understood. In this study, we collected and analyzed a large amount of gene expression data of different host cells infected with different subtypes of IAV. We found that IAV infection could affect host response and generate special differentially expressed genes in the distinct cell lines. And then we identified some upregulated genes (IFIT2, HERC5, and BATF2) that may exhibit antiviral activities in different cell types against IAV infection. Moreover, with the available data collections from distinct types of cells with different strains of IAV infection, we compared gene expression difference and the potential response difference of distinct types of cells to IAV stains.

Epithelial cells are the primary targets of IAV infection and can produce a protective environment by continuously secreting antiviral substances to initiate defense responses against infection. Many studies have shown that epithelial cells could produce many host cellular restriction factors in response to IAV infection (42). We summarized gene expression profiles of different human epithelial cells including human lung epithelial cells (A549), human airway epithelial cells (Calu-3), primary human bronchial epithelial cells (HBEC), well-differentiated human bronchial epithelial cells (wd-NHBE), human primary airway epithelial cells, human type I-like alveolar epithelial cells, and human type II-like alveolar epithelial cells. The results showed that various subtypes of IAV infection affected the amount of differentially expression genes, mainly involved in the interferon signaling pathway and cytokine and chemokine signaling pathway in different epithelial cells. But some overlapped genes (IFIT2, HERC5, and BATF2) were found to upregulate during different subtypes of IAV infection.

Interferon-induced protein with tetratricopeptide repeats 2 (IFIT2) is an interferon-stimulated gene (ISG) with a possible RNA-binding capacity and acts as an important restriction factor for many viruses, including rabies virus, Sendai virus, mouse hepatitis virus, hepatitis B virus, West Nile virus, and influenza virus (43–48). However, recent genome-wide knockout screens provided a novel proviral function of IFIT2, and knockout of IFIT2 remarkedly reduced diverse influenza viruses infection by decreasing the translational efficiency for viral mRNA and IFIT2-bound mRNAs. The influenza virus hijacked IFIT2 to preferentially bind viral mRNAs and prevent ribosome pausing for increasing viral replication (49).

HERC5, an interferon-induced HECT E3 enzyme was identified to upregulate in distinct cell types infected with different subtypes of IAV and might potentially play an important role for IAV replication. Existing research has revealed the antiviral activity of HERC5 against IAV; knockdown of HERC5 weakens IFN-beta-induced antiviral activities against IAV. HERC5 activated the ISGylation system by catalyzing conjugation of ISG15 onto IAV-NS1 proteins, leading to ISG15 modification of NS1 protein and blocking of the nuclear import of the NS1A protein (50, 51). In addition, our result that HERC5 acts as a key antiviral factor in IAV infection was in accordance with the result of a previous report, which identified HERC5 as a potential novel biomarker for the treatment of IAV by employing weighted gene co-expression network analysis (WGCNA) (52). Moreover, we found that although some microarray platforms did not include the BATF2 gene owing to the design of the array, the mRNA expression of BATF2, a member of AP-1 family transcription factor (53), was still significantly upregulated in multiple cells infected with different subtypes of IAV. BATF2 was proved as an antibacterial gene and was able to induce inflammatory responses in lipopolysaccharides and mycobacterium tuberculosis infection (54). IFNγ induced high levels of BATF2 mRNA expression to downregulate *trypanosoma cruzi*-induced IL-23 production in innate immune cells by blocking the recruitment of the c-JUN-ATF-2 heterodimer to the IL23a promoter and preventing the formation of the c-JUN-ATF-2 complex, and IFN-γ–induced BATF2 expression plays a key role for controlling Th17-mediagted immune responses during *trypanosoma cruzi* infection (54). In addition, BATF2 was broadly highly expressed in multiple tissues, including the spleen, lung, small intestine, cecum, and large intestine, and IFN-γ–induced BATF2 also disturbed T cell-mediated intestinal inflammation through the regulation of the IL-23/IL-17 axis that was associated with intestine inflammation (55). Previous studies reported that BATF2 was a proapoptotic gene and overexpression of BATF2 could lead to inhibition of DNA binding activation protein (AP1), which causes growth inhibition and induces apoptosis particularly in cancerous cells (53). Additionally, BATF2 could dephosphorylate phosphor-STAT3 to promote DUSP2 expression and upregulate of NF-κB activity, and could also be modified by N6-methyladenosine (m6A) to suppress its expression (56, 57). During feline infectious peritonitis virus (FIPV) infection, BATF2 showed continuous high expression and might be an important regulation factor of the death stages of infected cells (58). Our transcriptome analysis also showed that BATF2 was significantly increased in distinct cell types with IAV stains infection, indicating that it will be increasingly interesting to illustrate the role of BATF2 during IAV infection.

In conclusion, we demonstrated the gene expression pattern and molecular responses of distinct cells types among different subtypes of IAV infection. In general, IAV strains triggered a similar defense response among distinct cells types *via* the production of various antiviral cytokines and interferon-related genes, although few overlapped genes were present in distinct cells types. We identified that IFIT2, HERC5, and BATF2 might act as key antiviral factors to regulate IAV infection, but the molecular regulatory mechanisms of IFIT2, HERC5, and BATF2 involved in IAV infection still need to be validated.

## Data Availability Statement

The datasets presented in this study can be found in online repositories. The names of the repository/repositories and accession number(s) can be found in the article/**Supplementary Material**.

## Author Contributions

All authors contributed to the article and approved the submitted version. AZ designed this project, analyzed and interpreted data, and contributed to the writing of the manuscript. XD collected the data and modified the manuscript. BT analyzed and interpreted the data. ML drew the figures. BT and AZ supervised the financial support.

## Funding

This work was supported by the Key Lab of Process Analysis and Control of Sichuan Universities (No. 2018001). The project was sponsored by Sichuan Province for ROCS (0903/00021728) and NSFC (81902073).

## Conflict of Interest

The authors declare that the research was conducted in the absence of any commercial or financial relationships that could be construed as a potential conflict of interest.
